# Spontaneous Resolution of a Fetal Dural Sinus Thrombosis: One Case Report and Review of the Literatures

**Published:** 2012-03-20

**Authors:** Jinsong Gao, Juntao Liu, Xiya Zhou, Xuming Bian, Qing Dai, Feng Feng, Min Sheng, Chen Wang

**Affiliations:** 1 Department of Obstetrics and Gynecology,Peking Union Medical College Hospital, Beijing, China; 2 Department of Ultrasound (Qing Dai), Peking Union Medical College Hospital, Beijing, China; 3 Department of Radiology, Peking Union Medical College Hospital, Beijing, China; 4 Department of Pediatrics, Peking Union Medical College Hospital, Beijing, China

**Keywords:** Sinus Thrombosis, Intracranial, Fetus, Prenatal Diagnosis

## Abstract

Fetal dural sinus thrombosis is a rare finding. Most cases have been terminated without long-term follow-ups. Recently some reports have indicated the potentially favorable evolution of fetal dural sinus thrombosis. Most of the fetuses showing symptoms have been delivered with normal neurologic outcome. We report a case of fetal dural sinus thrombosis. Serial ultrasound and magnetic resonance images (MRI) showed the shrinkage of the thrombosis which indicated good prognosis. No physical or neurological abnormality was observed at 8-months follow-up. Conservative treatment is appropriate to prenatally diagnosed dural sinus thrombosis with favorable prognostic factors. Serial MRI or ultrasound should be taken every 1-2 months to monitor the thrombosis development and fetal well-beings.

## Introduction

Fetal dural sinus thrombosis is a rare finding because most pregnancies are terminated without a long-term follow-up. There are few reported cases and the prognosis is difficult to establish. Recently, there have been some reports of infants being born with normal neurological outcomes despite suffering from fetal dural sinus thrombosis, suggesting the favourable evolution of this disorder (1-6). Here we report a case of fetal dural sinus thrombosis diagnosed through ultrasound and magnetic resonance images (MRI), which resolved spontaneously and resulting in a pregnancy with normal neurological outcome. In this paper the literature has been reviewed to summarize the prenatal diagnosis, possible etiology, prognosis and related factors of fetal dural sinus thrombosis.

## Case report

A 29-year-old woman (gravida 1, papa 0) was referred to our hospital at 31 weeks of gestation because of a suspected presence of a posterior fossa cyst identified during a routine third-trimester ultrasound examination. The patient and her husband’s medical records were unremarkable. Maternal serum screening for Down’s syndrome in the second trimester represented low risk and the ultrasound at 12 and 20 weeks of gestation revealed no abnormalities.

**Fig 1:**
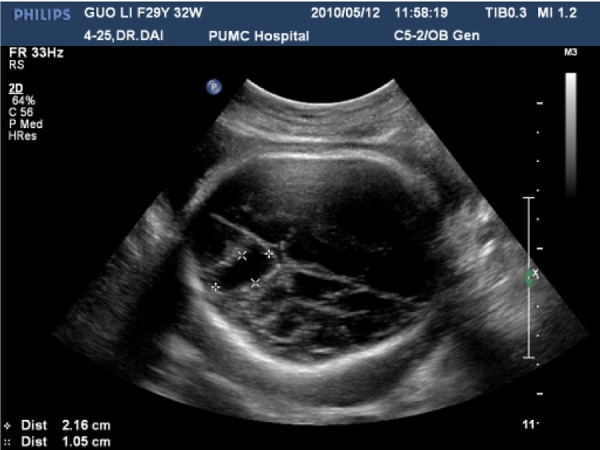
Ultrasound image (axial plane) of the fetal head at 32 weeks of gestation shows a cystic mass measuring 2.16×1.05cm at posterior fossa.

Ultrasound examination was repeated in our hospital and revealed a cystic mass measuring 2.16 × 1.05 cm in the posterior midline portion of the fetal brain (Fig 1). However, color Doppler showed no vascularity in or around the mass. There were no ventriculomegaly or other extracranial abnormalities, nor were there any signs of fetal hydrops or other anomalies.

**Fig 2:**
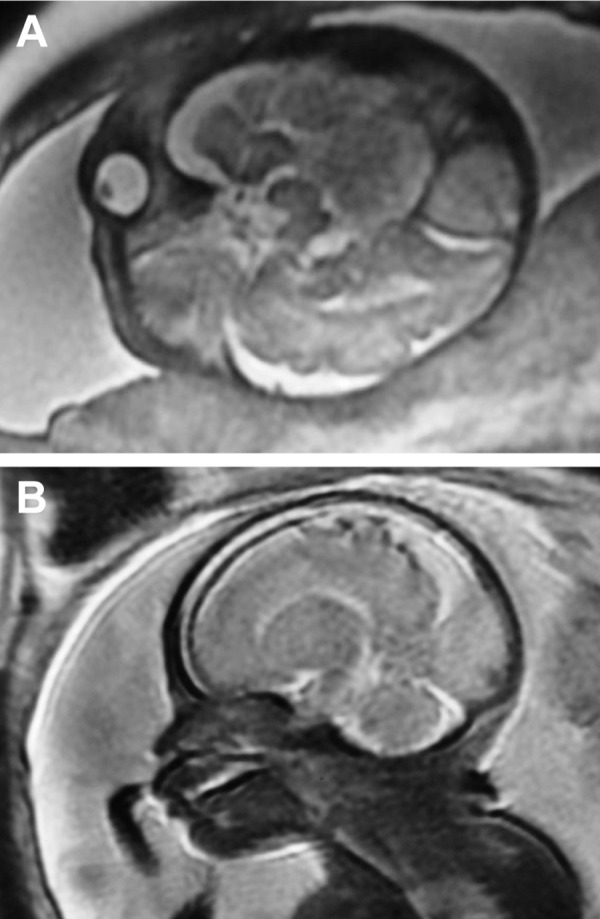
MRI images of the fetal head at 32 weeks’s gestation shows a triangle dural sinus thrombosis measuring 2.5×2.4cm at torcular. A. T2 weighted image at axial plane with high signal intensity, B. T2 weighted image at sagittal plane shows dilation of the superior longitudinal sinus.

Fetal magnetic resonance imaging (MRI was recommended to get more information. MRI at 32 weeks gestation showed a isolated triangle occipital mass measuring 2.5 × 2.4cm along the posterior surface of the brain with high signal intensity on T2 weighted MRI with occipital lobe compressed forward and superior longitudinal sinuses dilated (Fig 2). A fetal dural sinus thrombosis at torcular was suggested.

**Fig 3:**
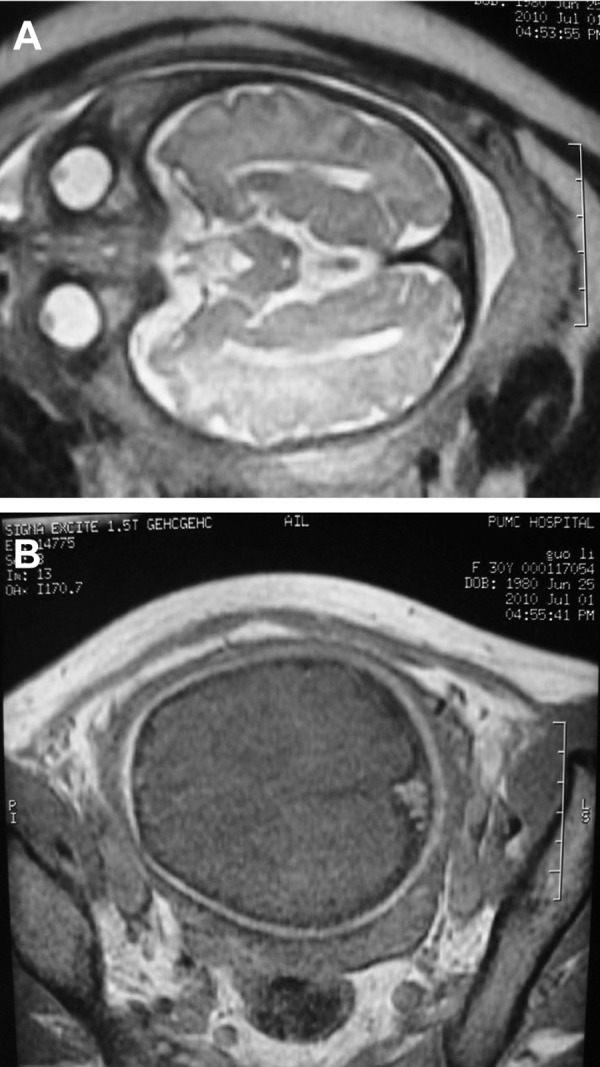
MRI images of the fetal head at 39 weeks’s gestation shows the torcular thrombosis becomes smaller than before. A. T2 weighted images at axial plane, B. T1 weighted image at axial plane.

**Fig 4:**
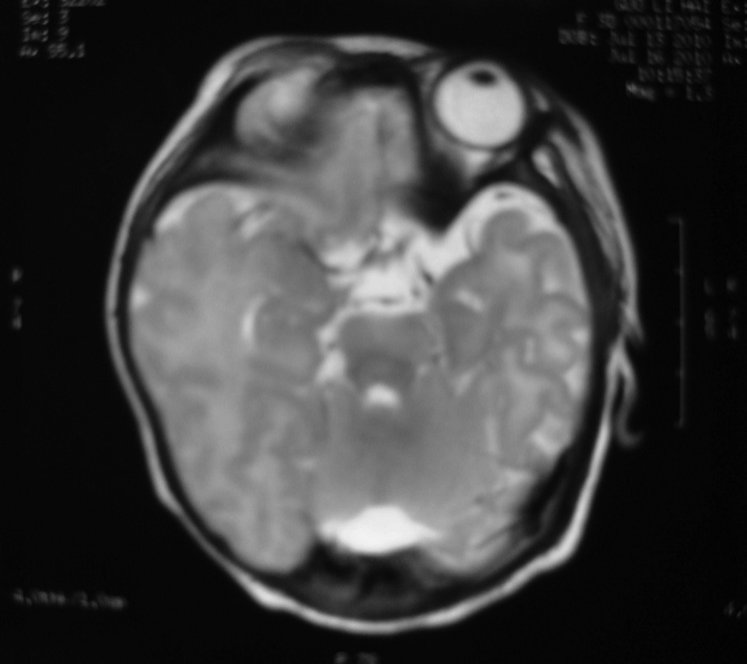
MRI images of neonate head three days after cesarean delivery shows further shrinkage of the torcular thrombosis (T2 weighted images at axial plane).

After a genetic counseling session where all the possible fetal outcomes were explained fully to the parents, they decided to continue the pregnancy. At 39 weeks of gestation, fetal MRI was repeated which showed the thrombosis had shrunk significantly without causing any brain compression (Fig 3). There were no complications encountered during the pregnancy and the baby was born via a Cesarean section at 40 + 2/7 weeks of gestation. Apgar scores were 10 at 1 minute and 5 minutes after birth. The female neonate was referred to NICU for further observation. The neonate weighted 3450 g and her head circumference was 35.2 cm which was within the normal range. No abnormalities were found during the physical exam. There was no evidence of hypercoagulability (plasma prothrombin time (PT) and INR, activated partial thromboplastin time (APTT) and APTT-R, d-dimer, Protein C, Protein S, anticardiolipin were all within the normal range). Three days after birth, MRI and MRV was performed which showed a further shrinkage of the thrombus (Fig 4) and the cerebral venous drainage was not compromised. The infant was discharged 7 days after birth and no physical or neurological abnormalities were observed at 8-months during a follow-up.

## Discussion

Thrombosis of the dural sinuses most often affects young adults, children and infants (7, 8) but can occasionally occur antenatally in uncomplicated pregnancies (2). Thrombosis of fetal dural sinus is very rare; only 13 cases have been reported in the literature (1-6). Because the sonographic features mimic those of an intracranial tumor, some unreported cases exist which if added to the reported cases, increases the incidence rate of this disorder. Unlike neonatal dural sinus thrombosis, no male dominance pattern can be detected for fetal dural sinus thrombosis (5).

So far the cause of fetal dural sinuses thrombosis hasn’t been identified. Trauma, thrombophilia, or dural sinus malformation have been related to dural sinuses thrombosis in infants (9,10). Although these factors can cause neonatal dural sinus thrombosis, they are absent in most prenatally diagnosed cases. Including this case, in the 14 reported cases , no cause was identified except in a case with hemangioma and one with suspected dural sinus malformation (DSM) (6). Fetal malformation, congenital heart defect, or thrombophilia were not detected in any of the reported cases. Therefore there may be different causes for fetal dural sinus thrombosis compared to neonatal and infant.

Most cases of fetal dural sinus thrombosis were diagnosed using ultrasound imaging and confirmed usingMRI (5). Real-time ultrasound associated with color Doppler imaging is the key factor in the prenatal diagnosis of dural sinus thrombosis (2). The typical ultrasound presentation is a round or triangle heterogeneous mass with a clear margin at the posterior fossa and an absence of blood flow inside the mass. Visualization of interruption of blood flow in the thrombosed sinus or an enlarged interhemispheric space corresponding to the dilated superior longitudinal sinus can also be found in some cases (6). Importantly, coexistent abnormalities, fetal hydrops suggesting fetal heart failure should be excluded during ultrasound examination. Dural sinus thrombosis should also be differentiated with intracranial tumor, cyst, hemorrhage and vein of Galen aneurysm (11, 12). A brain tumor is a cyctic or solid mass usually with mass-occupying effect as there is a change in the shape or size of normal anatomical structures. Prognosis of brain tumor is poor with affected neonates frequently dying shortly after birth (11, 13). MRI can provide additional information to evaluate fetal brain mass detected during an ultrasound, and is useful to distinguish thrombosis from brain tumor and other abnormalities (14). Sometimes, when the thrombus is large, nearby brain structures will be compressed (6).

Possible prognosis of fetal dural sinus thrombosis has crucial influence on prenatal consultation and decision-making. Of the 14 cases, 4 pregnanies terminated, 10 were live birth with 6 vaginal and 4 cesarean delivery. Two infants died postnatally, one of which died of DSM progression (6), another died of bleeding complications during operation. Of the 8 surviving infants the prognosis was good over a follow-up of 7-48 months. These patients had some common features including: normal pregnancy without malformation; normal brain structure without ventriculomegaly or brain infarction; no cardiac failure [indicative of dural arteriovenous shunt(DAVS)] or DSM (which is the typical type of neonate DAVS). In the cases reported although some of the thrombosis was enlarge at first, it shrank in size or even resolved completely in late pregnancy or after birth. These features suggest there is no coexistent abnormality and the cerebral venous drainage is not compromised thus achieves normal brain development. Torcular herophili is the most affected site which is present in 12/14 cases, 9/10 living birth and 7/8 surviving infants. Therefore, neither the size of the clot nor the surrounding dilation seems to be the predictive factor which is different from the situation in neonates and children.

On the base of available evidences, we suggest that conservative treatment is appropriate to prenatal diagnosed dural sinus thrombosis with favorable prognostic factors mentioned above. Serial MRI or ultrasound should be taken every 1-2 months to monitor the thrombosis development and fetal well-beings.

## Conflict of interest

All authors have no conflict of interest regarding this paper.
